# Diverse Mechanisms of Sp1-Dependent Transcriptional Regulation Potentially Involved in the Adaptive Response of Cancer Cells to Oxygen-Deficient Conditions

**DOI:** 10.3390/cancers8010002

**Published:** 2015-12-23

**Authors:** Shiro Koizume, Yohei Miyagi

**Affiliations:** Molecular Pathology & Genetics Division, Kanagawa Cancer Center Research Institute, 2-3-2 Nakao, Asahi-ku, Yokohama 241-8515, Japan; miyagi@gancen.asahi.yokohama.jp

**Keywords:** cancer, hypoxia, Sp1, transcriptional regulation

## Abstract

The inside of a tumor often contains a hypoxic area caused by a limited supply of molecular oxygen due to aberrant vasculature. Hypoxia-inducible factors (HIFs) are major transcription factors that are required for cancer cells to adapt to such stress conditions. HIFs, complexed with the aryl hydrocarbon receptor nuclear translocator, bind to and activate target genes as enhancers of transcription. In addition to this common mechanism, the induction of the unfolded protein response and mTOR signaling in response to endoplasmic reticulum stress is also known to be involved in the adaptation to hypoxia conditions. Sp1 is a ubiquitously-expressed transcription factor that plays a vital role in the regulation of numerous genes required for normal cell function. In addition to the well-characterized stress response mechanisms described above, increasing experimental evidence suggests that Sp1 and HIFs collaborate to drive gene expression in cancer cells in response to hypoxia, thereby regulating additional adaptive responses to cellular oxygen deficiency. However, these characteristics of Sp1 and their biological merits have not been summarized. In this review, we will discuss the diverse mechanisms of transcriptional regulation by Sp1 and their potential involvement in the adaptive response of cancer cells to hypoxic tumor microenvironments.

## 1. Introduction

Cancer cells within the solid tumor microenvironment are commonly exposed to conditions of reduced oxygen tension, called hypoxia, as a result of inefficient and disordered vascularization compared to normal tissues. Tissue hypoxia is generally defined as a low oxygen concentration of less than 2% [1,2], and very severe hypoxia conditions (≤0.1% O_2_) can exist within tumor tissues [1,2]. This environment should be disadvantageous for the efficient growth of cancer cells given the insufficient supply of not only O_2_, but also other serum components, including nutrients; however, cancer cells use various means to overcome these stress conditions [1,2]. For example, expression of the transcription factors hypoxia-inducible factor 1α (HIF-1α) and hypoxia-inducible factor 2α (HIF-2α; also known as EPAS-1) in cells under hypoxia is known to be involved in this stress response [3,4]. HIFs are expressed in normal cells under physiological hypoxic conditions [3]; HIF-1α is ubiquitously expressed, whereas HIF-2α exhibits tissue selectivity [3]. Both HIFs can form a heterodimer complex with constitutively-expressed aryl hydrocarbon receptor nuclear translocator (ARNT; also known as HIF-1β) and, thereby, occupy nucleotide sequences called hypoxia response elements (HREs) within target gene loci [3,4]. The HIF-ARNT complex can act as an enhancer of transcription with coactivators, such as histone acetyltransferase p300 [3,4]. This conventional mechanism is believed to regulate key genes required for survival under poorly-vascularized harsh microenvironments [1].

The chronic hypoxia status of tumor microenvironments might be dynamically changing given the variations in vascularity and stromal components [5,6]. There are also acute modes of tumor hypoxia known as intermittent or cycling hypoxia, which may further affect the tumor microenvironment by producing reactive oxygen species [5]. In addition, there must be variable nutrient, growth factor and hormone supplies related to different conditions of hypoxia within tumor tissues. Thus, multiple molecular mechanisms for regulating the adaptive response to hypoxia may exist depending on the heterogeneous conditions of the tissue oxygenation status. Indeed, in addition to HIF-ARNT-driven mechanisms, a variety of stress response mechanisms exists within the tumor microenvironment. For example, induction of the unfolded protein response (UPR) and mammalian target of rapamycin (mTOR) signaling in response to endoplasmic reticulum (ER) stress are well-established cellular responses that are involved in the adaptation to severe hypoxia [7]. These mechanisms can activate additional genes required for tumor survival and growth by inducing downstream transcription factors [6].

Specificity protein 1 (Sp1) was identified as a transcription factor that binds to the SV40 early promoter [8,9]. This protein is a member of the family of Sp/Krüppel-like factors and is a well-characterized transcription factor involved in the basal regulation of a number of genes, including housekeeping genes [8,9]. Sp1 typically binds GC-rich double-stranded DNA via zinc fingers and recruits the general transcription machinery to the target gene promoter regions; thus, Sp1 is essential for physiological cell function [8,9]. There is increasing experimental evidence that the function of Sp1 can be regulated post-translationally by multiple means [8,9]. Such modulated Sp1 may play roles in gene expression required for the expression of malignant phenotypes in cancer cells [9]. Recently, accumulating experimental evidence suggests that Sp1 may be involved in inducible gene expression in response to hypoxia, thus contributing to tumor biology. However, in contrast to HRE-dependent mechanisms, how and to what extent these Sp1-dependent mechanisms contribute to hypoxia-driven gene transcription in cancer cells is not fully understood. In this review, we will discuss the current understanding of Sp1 function as a mediator of transcriptional induction in response to various hypoxia-related stimuli and its potential biological roles, primarily focusing on the expression of malignant phenotypes. Some Sp1-driven transcriptional induction mechanisms that are not necessarily associated with hypoxia or cancer cells will also be discussed in relation to their potential involvement in the adaptation of cancer cells to an oxygen-deficient tumor microenvironment.

## 2. Diverse Mechanisms of Sp1-Mediated Transcriptional Activation Potentially Occur under Hypoxia

### 2.1. Sp1 Collaborates with HIFs to Enhance HRE-Driven Transcriptional Activation

**Figure 1:**
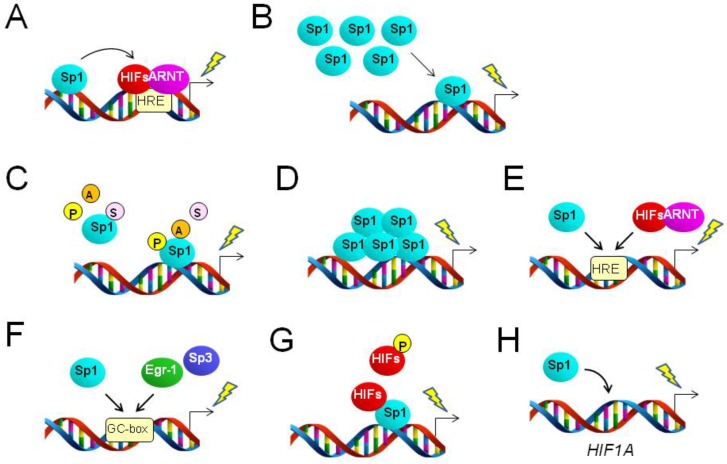
Various mechanisms of Sp1-driven activation of gene promoter regions in response to hypoxia. Bent arrows and lightning symbols indicate the transcription start site and transcriptional activation, respectively. (**A**) Sp1 augments hypoxia response element (HRE)-dependent gene expression; (**B**) increased expression level enhances Sp1 binding to gene promoters, leading to transcriptional activation; (**C**) post-translational modifications affect the activity of Sp1, resulting in Sp1-dependent transcriptional regulation. P and A in the circles indicate phosphorylation and acetylation, respectively, potentially resulting in the activation of Sp1. S in circles designates SUMOylation, which can downregulate Sp1 binding. (**D**) Self-assembly of Sp1 on the promoter region can activate downstream target genes; (**E**) Sp1 and the HIF-ARNT complex compete for binding to the HRE to regulate target genes; (**F**) Sp1, Egr-1 and Sp3 compete for binding to the GC-box to regulate target genes; (**G**) direct association or dissociation between Sp1 and HIFs regulates target genes. Phosphorylation of HIFs is involved in this process as an inhibitory modification of Sp1-HIF interactions. (**H**) Sp1 directly or indirectly targets the *HIF1A* gene to increase its transcript level.

The typical mechanism of transcriptional activation by HIFs involves the enhancement of the basal promoter activity of target genes. The HIF-ARNT complex can occupy the enhancer region proximal or distal to the core promoter region of multiple genes, leading to increased transcriptional activities [3]. There are Sp1 binding sites near the known HRE region in various genes, and Sp1 binding is required for the full activity of the HRE within gene-regulatory regions (Figure 1A). Hypoxia-driven expression of multidrug resistance (*MDR1*, formally *ABCB1*) [10], retinoic acid receptor-related orphan receptor α4 (*RORA*) [11], basigin (*CD147*, formally *BSG*) [12], carbonic anhydrase 9 (*CA9*) [13,14] and glyceraldehyde-3-phosphate dehydrogenase (*GAPDH*) [15] gene loci falls into this mechanistic category, at least in some cancer cell types. HRE and Sp1 sites were found to collaborate to enhance the promoter activity of these genes in a HIF-1α-dependent manner in several types of cancer cell exposed to hypoxia or hypoxia mimetic stimuli [10,11,12,13,14,15]. Induction of *CA9* seems to be further enhanced when cells are exposed to both hypoxia and high cell density conditions [13]. The mechanisms described above are typical examples of the collaboration of Sp1 with HIFs in the activation of genes potentially involved in the adaptation of cancer cells to hypoxia. Furthermore, it was shown that HIF-1α expression is increased in the human breast cancer cell line MDA-MB-231 in response to insulin exposure, resulting in nuclear accumulation of this transcription factor. HIF-1α in the nucleus can cooperate with Sp1 to activate the leptin (*LEP*) gene promoter region via potential Sp1 and HIF-1α binding sites [16]. However, it is unclear whether this mechanism is applicable to hypoxia-driven *LEP* induction in cancer cells.

In addition, HIF-2α binding collaborates with proximal Sp1 to induce gene expression in normal and cancer cells. Studies have revealed HRE sites within the promoter regions of the ATPase copper-transporting, alpha polypeptide (*ATP7A*) gene in rat epithelial cells [17] and the plasminogen activator receptor-1 (*PAI1*, formerly *SERPINE1*) gene in the human lung adenocarcinoma cell line A549 [18]. These genes can be activated in response to iron deficiency [17] or hypoxia [18] via the cooperation of Sp1 with proximal HIF-2α, presumably via the HRE. Moreover, this collaboration can also be applied to the induction of the HRE-containing MT1-MMP (*MMP14*) gene in kidney cancer cells, in which the function of von Hippel-Lindau (VHL) protein is lost, and therefore, HIFs are expressed even under normoxia [19].

### 2.2. Altered Expression Level of Sp1 Regulates Target Genes under Hypoxia

Hypoxia-driven expression of Sp1 can be regulated transcriptionally and translationally in various human cells and, thus, contributes to downstream gene expression (Figure 1B). It was shown that the Sp1 protein level is increased in neuronal cells exposed to ischemic conditions, which are deficient in both O_2_ and glucose [20]. Reactive oxygen species produced under this severe hypoxia condition activate the translation of Sp1 protein via an internal ribosome entry site in the 5′-untranslated region of *Sp1* mRNA [20]. This upregulation of the *SP1* gene is further enhanced at the transcriptional level through autonomous feed-forward activation by the synthesized Sp1. HIF-1α-driven expression of the *SP1* gene has also been reported in the regulation of the prion protein (*PRNP*) gene during neurotoxicity in human cells [21], although it is unclear whether this transcriptional activation is HRE dependent. It is also currently unknown whether these mechanisms can contribute to human malignancies.

Hypoxia-mediated upregulation of Sp1 induces the expression of the metallopeptidase domain 17 (*ADAM17*) gene in the human glioma cell line U87, although the detailed mechanisms of this Sp1 induction are unclear [22]. The human endosialin (*CD248*) gene can be activated in some normal and cancer cells [23,24] via binding of Sp1 to the promoter region under high cell density culture conditions [23]. The authors of the study implicated that Sp1 protein may be increased in response to this potential mild hypoxia condition [13] via transcriptional activation of the *SP1* gene by the Ets-1 transcription factor, as the gene encoding Ets-1 is a potential HIF-1α target [23].

A recent study has shown that multiple HRE sites exist within the rat *SP1* promoter region [25]. HIF-1α binds these HREs, thereby increasing Sp1 protein levels in conditions of cerebral ischemia. This increase in Sp1 protein may enhance the transcription of the target sulfonylurea receptor 1 (*ABCC8*) gene under ischemic conditions [25]. However, it is unclear whether this mechanism is applicable to the promoter region of the human *SP1* gene in cancer cells.

HIF-2α may also contribute to increased Sp1 levels depending on the cell type. To date, there is a report showing that the activity of Sp1 can be increased by HIF-2α to augment the expression of the interleukin-8 (*CXCL8*, formerly *IL8*) gene in human endothelial cells [26]. It was shown that the relative activity of Sp1 in myocytes increased as the expression of Sp3, which could compete with Sp1 for the same DNA binding site, is decreased in response to hypoxia [27]. This relative abundance of Sp1 over Sp3 can activate enolase 3 (*ENO3*) and pyruvate kinase-M (*PKM*) genes under hypoxia [27]. So far, it is unclear whether these types of Sp1 activation occur and perform any function in cancer cells.

### 2.3. Altered Activity of Sp1 Regulates Target Genes under Hypoxia

Phosphorylation is a hallmark of the active state of Sp1 protein [8,9]. Several studies have demonstrated that multiple intracellular signaling mechanisms under hypoxia are associated with the phosphorylation status of Sp1 protein. This post-translational modification regulates Sp1 activity to occupy target double-stranded DNA sequences (Figure 1C). Indeed, in breast cancer cells exposed to hypoxia, p53 protein was found to suppress Sp1 activity via inhibition of Src kinase activity, resulting in the downregulation of the vascular endothelial growth factor (*VEGF*) gene [28]. In contrast, the Akt-PI3K axis positively regulates *VEGF* expression under hypoxia via Sp1 activation in Hep3B liver cancer cells [29], SQ20B head and neck cancer cells [30] and A549 lung adenocarcinoma epithelial cells [29]. This signaling cascade also correlates with hypoxic activation of the NADP+-dependent isocitrate dehydrogenase (*IDH2*) gene in the PC3 prostate cancer cell line [31]. Indeed, phosphorylation of Sp1 was demonstrated in SQ20B and A549 cells [30]. It was also shown that hypoxia and the reoxygenation-driven regulation of Syk-Lck cross-talk could enhance the translocation of Sp1 into nuclei, leading to increased expression of target genes, such as urokinase-type plasminogen activator (*PLAU*) and matrix metalloproteinase-9 (*MMP9*) in MCF-7 breast cancer cells [32]. Furthermore, the monocyte chemoattractant protein-1 (*CCL2*) gene was found to be activated in melanoma cells during exposure to hypoxia and the reoxygenation process [33]. It was shown that Sp1 and NFκB become activated under this hypoxia-associated condition, thereby promoting binding of Sp1 to the *CCL2* promoter region to activate gene expression [33]. The urokinase plasminogen activator receptor (*PLAUR*) gene was found to be activated in response to hypoxia in a Bcl-2 protein-dependent manner [34]. It was further shown that Bcl-2 induces ERK signaling, which increases the amount and activity of Sp1, thereby activating the downstream *PLAUR* gene [34].

Hypoxic induction of the tenascin-X (*TNXB*) gene in the breast cancer cell line MCF-7 involves another type of Sp1 activation mechanism. Histone deacetylase-1, a known negative regulator of chromatin, binds to Sp1 associated with the promoter of the *TNXB* gene, resulting in the suppression of this gene under normoxia [35]. However, in response to hypoxia, *TNXB* becomes activated via dissociation of histone deacetylase-1 from Sp1 [35], suggesting that the protein acetylation process is involved in this Sp1-dependent *TNXB* gene activation (Figure 1C). Furthermore, SUMOylation (Figure 1C) by PIASy can modify Sp1 and suppress the expression of the sirtuin 1 (*SIRT1*) gene in ovarian [36] and lung [37] cancer cells.

Unlike the HIF-independent regulation of Sp1 activity described above, it is also possible that HIF-1α negatively regulates Sp1 activity in cancer cells [38]. RNA interference-mediated inhibition of HIF-1α expression induced by the iron-depriving agent desferrioxamine increases Sp1 binding to the *CDKN1A* promoter region in cancer cells [38].

Post-transcriptional modifications other than phosphorylation may enhance the activity of Sp1 in cancer cells exposed to hypoxia. Sp1 proteins can associate with each other on gene promoter regions, thereby synergistically activating target genes [39]. It has been reported that Sp1 can be cross-linked by increased transglutaminase expression in neuronal cell nuclei under conditions of brain ischemia [40]. Induction of this transglutaminase expression occurs through the unfolded protein response (UPR), a mechanism that is activated in severe hypoxic tumor microenvironments. This self-assembled Sp1 on the gene promoter region is active enough to augment the expression of downstream target genes (Figure 1D), such as the adenylate cyclase-activating polypeptide receptor (*PAC1*). However, to date, there are no reports indicating that self-assembly of Sp1 occurs in tumors and contributes to the expression of cancer phenotypes.

### 2.4. Sp1 Shares Binding Sites with Other Transcription Factors to Regulate Hypoxia-Driven Gene Expression

The HRE is not strictly restricted to a specific DNA sequence, but can be represented as 5′-(G/C/T)ACGTGC(G/C)-3′ or 5′-RCGTGC-3′ [41]. Thus, HIFs can occasionally share their binding sites with Sp1 within some gene-regulatory regions, enabling Sp1 to participate in gene expression via HREs (Figure 1E). It was previously reported that HIF-1α is induced in keratinocytes exposed to ultraviolet B irradiation [42]. HIF-1α induced under this non-hypoxic condition binds HREs similar to Sp1 consensus sequences within the regulatory region of the nucleotide excision repair genes *XPC* and *XPD* (formerly *ERCC2*), thereby increasing their expression [42]. The GC-box-like HRE sequences could be predominantly occupied by constitutively-expressed Sp1 under early irradiation conditions, when HIF-1α is degraded by the proteasome [42]; however, HIF-1α rather than Sp1 is responsible for the induction of these genes under long-term ultraviolet B exposure.

Furthermore, some transcription factors could share GC-rich binding sites with Sp1 [43] to regulate gene expression in response to hypoxia (Figure 1F). Egr-1 is known to be upregulated in response to hypoxia in cancer cells and competes with Sp1 for binding to the GC-box [43]. Thus, Egr-1 could mediate hypoxia-driven expression of target genes via Sp1 binding sites. In this case, Sp1 negatively contributes to Egr-1-driven gene expression. Typical examples are Egr-1-mediated transcriptional induction of tissue factor (*F3*) and N-myc downregulated gene 1 (*NDRG1*). The increased Egr-1 produced in cells in response to hypoxia binds promoter regions via GC-rich sequences, which potentially share a Sp1 binding site [43,44]. It is likely that intracellular signaling associated with protein kinase C-β, but not HIF signaling, is involved in hypoxic induction of Egr-1 [45].

Sp3 also shares DNA elements with Sp1 (Figure 1F). It was reported that Sp3 competes with Sp1 to negatively regulate *ENO3* and *PKM* genes under hypoxia, as described above (see Section 2.2).

### 2.5. Direct or Indirect Interaction between Sp1 and HIFs Regulates Gene Expression

In U-2 OS osteosarcoma cells, HIF-1α can directly associate with Sp1 via its N-terminal PAS domain region, whereas Sp1 interaction with HIF-2α is prevented because the threonine 324 residue within the PAS domain is phosphorylated by protein kinase D1 [46,47]. This HIF-Sp1 complex associated with the promoter region (Figure 1G) negatively regulates the transcription of DNA mismatch repair genes in cancer cells [46,47]. Transcriptional activation of the mutS homolog (MSH) genes (*MSH2* and *MSH6*) is mediated by the Sp1-Myc complex on the promoter region. HIF-1α displaces Myc from Sp1 associated with the gene promoter region, leading to the repression of *MSH* gene expression [46]. On the other hand, the Sp1-HIF-1α interaction similarly suppresses the expression of cell cycle-related genes via inhibition of c-Myc binding to these genes in cancer cells. HIF-2α indirectly and oppositely functions in this mechanism by increasing the binding of c-Myc [48].

Activation of the *TNXB* gene in MCF-7 cells exposed to hypoxia also involves Sp1-HIF-1α interaction [35]. This protein-protein interaction presumably competes with the Sp1-HDAC1 interaction, thereby increasing *TNXB* expression via activation of Sp1 [35], as described in Section 2.2.

In contrast to the abrogated Sp1-HIF-2α interaction in some cell types, direct association between Sp1 and HIF-2α on gene promoters is also possible (Figure 1G) and activates multiple genes in ovarian clear cell carcinoma (CCC) cells [41,49,50]. Sp1 is responsible for basal expression of coagulation factor VII (*FVII*), intercellular adhesion molecule-1 (*ICAM1*), Krüppel-like factor 6 (*KLF6*) and jun proto-oncogene (*JUN*). HIF-2α induced in CCC cells in response to hypoxia might bind to gene promoter regions via Sp1 and augment the expression of these genes. This process is ARNT independent, possibly because the ARNT binding site within HIF-2α (PAS domains) would be occupied with Sp1 [49,50]. In addition, a recent report has shown that induction of the *FBI1* gene under hypoxia can be mediated by Sp1-HIF-2α interaction; thus, this transcriptional regulation may fall within this mechanistic category [51]. Furthermore, hypoxia-driven activation of *FVII*, *ICAM1*, *KLF6* and *JUN* genes, but not *VEGF*, can be synergistically enhanced when cells are exposed to both hypoxia and serum starvation conditions [49,52]. The mechanism of these synergistic gene activations does not involve the UPR [49,50]. In the case of *ICAM1*, *KLF6* and *JUN* genes, it was revealed that an insufficient supply of long chain fatty acids is a major cause of this synergism [49], and this process was found to involve mTOR [50]. Overall, the interaction between Sp1 and HIFs on the gene-regulatory regions in cancer cells seems to be regulated via differential control of PAS domain phosphorylation.

### 2.6. Sp1 Targets the HIF1A Gene, Potentially Contributing to Hypoxic Gene Expression

As previously described, Sp1 can cooperate with HIFs to regulate transcription. The human *HIF1A* gene encoding HIF-1α is also known to be a target of Sp1 [53,54,55], as the gene promoter region contains consensus Sp1 binding sequences [56]. Moreover, Sp1 directly affects the *HIF1A* gene [55]. Thus, in addition to increased protein level via inhibition of proteasomal degradation, HIF-1α expression can be regulated at the transcriptional level via direct or indirect interaction of Sp1 with the *HIF1A* promoter region in cancer cells (Figure 1H). Sp1 cooperates with p53 to indirectly downregulate the *HIF1A* gene in cancer cells [54]. In contrast, Sp1-driven *HIF1A* activation can be induced by echinomycin [56]. Treatment of cancer cells with this antibiotic augments Sp1 activity under normoxia, resulting in elevation of *HIF1A* transcript levels, potentially by increasing binding activity rather than by increasing the protein level [56]. However, echinomycin is also known to inhibit HIF-1α binding by intercalating into target double-stranded DNA under hypoxia to decrease the transcriptional activity of target genes [56,57]. The dual effect of this antibiotic on HIF-1α mRNA regulation may be dependent on cell type and/or environmental conditions. Furthermore, it was reported that negative regulation of the *HIF1A* gene by protein arginine methyltransferase 1 in cancer cells involves altered Sp1 protein expression level [58]. These effects should be inhibited by the treatment of cells with mithramycin A, which associates with GC-rich DNA sequences, thereby interfering with Sp1 binding [53]. To date, there are no reports on Sp1-mediated regulation of the *EPAS1* gene, which encodes HIF-2α, in cancer cells.

### 2.7. Other Relationships between Sp1 and HIFs as Mediators of Hypoxia-Driven Transcription

It is likely that Sp1-HIF interactions also regulate gene expression via molecular mechanisms that do not necessarily fall within the mechanistic categories shown in Figure 1. For example, it was reported that HIF-1α expression inversely correlates with Sp1 binding to the *CDKN1A* promoter region in cancer cells [38]. Specifically, HIF-1α can inhibit Sp1 binding, followed by downregulation of target genes (Section 2.3). Details of this Sp1-HIF-1α interaction are currently unclear. Furthermore, the *NDRG1* gene [43] encoding N-myc downstream-regulated 1 protein (also called Cap43) can be upregulated in renal cancer cells, in which VHL protein is absent, potentially via induction of HIFs [59]. This gene expression can be abrogated when the Sp1 binding site within the *NDRG1* promoter region is mutated. Thus, one possible explanation of this transcriptional induction is via Sp1-HIF interaction. However, this mechanism has not yet been convincingly demonstrated.

In tumor tissues, irradiated cancer cells can influence the characteristics of surrounding non-irradiated cells via a mechanism called the bystander effect [60]. Inverse transcriptional control between Sp1 and HIF-1α was reported between bystander-positive and -negative cells [60]. The detailed molecular mechanisms of this phenomenon are currently unclear; however, these results raise the possibility that local hypoxic conditions within tumors may determine which transcription factor(s) predominantly control transcription in the local tissue area.

HIF-2α plays a major role in hypoxia-driven expression of *FVII* and *ICAM1* genes in CCC cells [49,50]. HIF-1α also contributes to the expression of these genes. RNA interference-mediated silencing of HIF-1α decreased hypoxia-driven expression of these genes, as in the case of HIF-2α knockdown [49,50]. However, unlike HIF-2α, binding of HIF-1α to the promoter regions of these genes was not detected [49,50]. Moreover, these transcriptional activations are HRE independent [49,50]. Thus, the detailed molecular mechanisms of the contribution of HIF-1α to *FVII* and *ICAM1* gene expression in CCC cells are currently unclear, and HIF-1α may indirectly affect the activation of these genes.

## 3. Functions of Sp1-Regulated Hypoxia-Responsive Genes

### 3.1. The Sp1-Dependent Fraction of Hypoxia-Driven Transcriptional Activation Regulates Multiple Cellular Functions

Diverse functions of genes that can be activated in response to hypoxia in an Sp1-dependent manner have been reported to date, as summarized in Table 1. Thirty proteins, excluding RORA, XPC and ERCC2, are known to play important roles in the expression of malignant phenotypes involved in cancer initiation and/or progression, such as increased invasion, aberrant metabolism and enhanced survival. For example, cancer cells are known to undergo morphologic changes known as epithelial-to-mesenchymal transition (EMT) during their invasion and migration processes. Post-translational modification of Sp1 in response to hypoxia seems to enhance EMT via downregulation of the *SIRT1* gene [36,37].

It was reported that *RORA* expression has a suppressive effect on cancer progression [61]. Thus, the roles of the hypoxia-induced *RORA* gene product in cancer progression are currently unclear. Regulation of the nucleotide excision repair genes *XPC* and *ERCC2* contributes to cancer prevention rather than progression, because the function of these genes is to maintain genomic integrity. It is also unclear how the increase in *KLF6* and *JUN* gene products in response to hypoxia influences cancer phenotypes, as *KLF6* [62,63] and *JUN* [64,65] are known to act as oncogenes or tumor suppressors depending on various factors, such as cell type and stimuli derived from different cellular environments. *GAPDH* encodes a glycolytic enzyme and is considered a housekeeping gene. It has been revealed that the protein product of this gene plays multiple roles in cancer progression [66] (Table 1). Thus, it is clear that Sp1 plays multiple roles in the progression of cancers exposed to oxygen deficiency. However, conventional HRE-dependent gene expression is also known to be responsible for the expression of the same cancer phenotypes, as depicted in Table 1. Accordingly, the relative importance of the Sp1-dependent fraction of hypoxia-driven transcriptional induction is largely obscure. One plausible possibility is that Sp1 contributes to fine-tuning HRE activity for the appropriate adaptation of cancer cells to dynamically changing hypoxic conditions.

**Table 1:** List of genes that can be activated in response to hypoxia via the transcription factor Sp1. Mechanistic categories and potential effects on cancer cell biology for each gene are also depicted. * Official names from the HUGO Gene Nomenclature Committee (www.genenames.org). N/A: not applicable.

	Official Gene Symbol *	Official Full Protein Name *	Potential Mechanistic Category Shown in Figure 1	Possible Effect on Cancer Initiation or Progression	Phenotype References
1	*ABCB1 (MDR1)*	ATP-binding cassette, sub-family B (MDR/TAP), member 1	A	drug resistance	[10]
2	*RORA*	retinoic acid receptor-related orphan receptor A	A	suppressive to cancer phenotypes?	[61]
3	*BSG (CD147)*	basigin (Ok blood group)	A	invasiveness, survival	[12]
4	*GAPDH*	glyceraldehyde-3-phosphate dehydrogenase	A	glycolysis, drug resistance, cell proliferation, tumorigenesis	[66]
5	*CA9*	carbonic anhydrase IX	A	cellular pH control	[13,14]
6	*LEP*	leptin	A	invasiveness	[16]
7	*SERPINE1 (PAI1)*	serpin peptidase inhibitor, clade E, member 1	A	motility, invasiveness, angiogenesis	[18]
8	*MMP14*	matrix metallopeptidase 14 (membrane-inserted)	A	invasiveness	[19]
9	*ADAM17*	ADAM metallopeptidase domain 17	B	invasiveness	[22]
10	*CD248*	CD248 molecule, endosialin	B	tumor growth, invasiveness, metastasis	[23,24]
11	*ENO3*	enolase 3 (beta, muscle)	F	metabolism (glycolysis)	[67]
12	*PKM*	pyruvate kinase, muscle	F	metabolism (glycolysis)	[67,68]
13	*VEGFA*	vascular endothelial growth factor A	C	angiogenesis, radioresistance	[28,29,30,32]
14	*IDH2*	isocitrate dehydrogenase 2 (NADP+), mitochondrial	H	survival under various harmful effects such as ionizing radiation	[69]
15	*PLAU*	plasminogen activator, urokinase	C	angiogenesis	[32]
16	*MMP9*	matrix metallopeptidase 9	C	Invasiveness, angiogenesis, metastasis	[32]
17	*CCL2*	chemokine (C-C motif) ligand 2	B, C	regulation of tumor immune response	[33]
18	*PLAUR*	plasminogen activator, urokinase receptor	C	angiogenesis, invasiveness, motility	[34]
19	*TNXB*	tenascin XB	C, G	motility, invasiveness, drug resistance	[70,71]
20	*CDKN1A*	cyclin-dependent kinase inhibitor 1A	N/A	cell cycle progression	[38]
21	*SIRT1*	sirtuin 1	C	invasiveness	[36,37]
22	*XPC*	xeroderma pigmentosum, complementation group C	E	nucleotide excision repair upon UV irradiation	[42]
23	*ERCC2 (XPD)*	excision repair cross-complementation group 2	E	nucleotide excision repair upon UV irradiation	[42]
24	*F3*	coagulation factor III (tissue factor)	F	motility, invasiveness, hypercoagulation	[41,49,72]
25	*NDRG1*	N-myc downstream regulated 1	F	NR	[43]
26	*MSH2*	mutS homolog 2	G	mismatch repair, radioresistance	[46,73]
27	*MSH6*	mutS homolog 6	G	mismatch repair, radioresistance	[46,73]
28	*F7 (FVII)*	coagulation factor VII	G	motility, invasiveness, hypercoagulation	[41,49,72]
29	*ICAM1*	intercellular adhesion molecule 1	G	invasiveness, survival	[50]
30	*KLF6*	Krüppel-like factor 6	G	transcription factor act as oncogene or tumor suppressor	[62,63]
31	*JUN*	jun proto-oncogene	G	transcription factor act as oncogene or tumor suppressor	[64,65]
32	*ZBTB7A (FBI1)*	zinc finger and BTB domain containing 7A	G	survival	[51]
33	*HIF1A*	hypoxia inducible factor 1, alpha subunit	H	activation of HRE-dependent hypoxia responsive genes	[53,54,55,56]

### 3.2. Sp1-Dependent Fraction of Hypoxia-Driven Transcriptional Activation Contributes to Radioresistance of Cancer Cells

Tissue hypoxia is a major cause of resistance of cancer cells to radiation therapy because of the low level of reactive oxygen species derived from molecular oxygen [73]. So far, multiple studies have shown that the Sp1-dependent fraction of hypoxia-driven transcriptional activation is associated with the radioresistance of cancer cells. However, DNA repair activities in those hypoxic cancer cells are diminished, potentially leading to genomic instability [5,73]. Thus, why hypoxic cancer cells are radioresistant despite impaired DNA integrity remains a subject of debate. Indeed, the expression of the mismatch repair genes *MSH2* and *MSH6* is suppressed in an Sp1-dependent manner in cancer cells exposed to hypoxia [46,73] (Table 1), suggesting that Sp1-driven gene expression in response to hypoxia may contribute to the radioresistance of cancer cells.

Pharmacologic inhibition of Sp1-driven induction of the *VEGF* gene under hypoxia may increase the radiosensitivity of cancer tissues. It was shown that treatment of cancer cells with the small-molecule inhibitor nelfinavir suppresses the activity of the intracellular signaling pathway. This downregulation is followed by Sp1 inactivation, resulting in a reduction of *VEGF* expression, which appears to correlate with improved tissue radiosensitivity [30] by suppressing the highly deregulated and increased vascularity. Increasing experimental evidence has shown that mutated *IDH2* plays roles in malignancy under hypoxia by mediating the production of an oncometabolite, 2-hydroxyglutarate, from α-ketoglutarate [69]. It has also been shown that wild-type *IDH2* expression contributes to the resistance of cancer cells to programmed cell death caused by various environmental insults, including ionizing radiation (Table 1). This process should be mediated via glutathione metabolism [69]. Thus, Sp1-mediated overexpression of the wild-type *IDH2* gene in response to hypoxia potentially contributes to the radioresistance of cancer cells.

### 3.3. Sp1 May Contribute to Sensing the Characteristics of Hypoxic Cancer Tissues that Are Insufficiently Supplied with Serum Long Chain Fatty Acids

An additional function of Sp1 is suggested by the findings of a recent study showing synergistic transcriptional induction under serum starvation and hypoxia (SSH) conditions. This study revealed that transcriptional induction of *ICAM1* and multiple other genes is relatively weak when CCC cells are exposed to either hypoxia or serum starvation [50]. However, these genes, and particularly *ICAM1*, are dramatically activated when cells are cultured under SSH conditions. Importantly, Sp1 is involved in this process, as described in Section 2.5 and Figure 1G. This study further demonstrated that addition of long chain fatty acids (LCFA) associated with albumin suppresses this highly synergistic gene expression. These results suggest that the absence of LCFA is responsible for the synergistic activation of genes under SSH. Histochemical studies using a xenograft CCC tumor model revealed the presence of two hypoxic areas within the same tumor: hypoxic tissue with high levels of neutral lipids and hypoxic tissue with low levels of neutral lipids [50]. Thus, one intriguing possibility regarding the relative importance of Sp1-dependent compared to HRE-dependent mechanisms is that Sp1-mediated gene expression might help to discriminate the severity of tissue hypoxia depending on tissue lipid levels.

## 4. Future Perspectives, Including Clinical Implications

Transcriptional activation mediated via HIF-ARNT complex formation and its subsequent binding to HRE is well established. In contrast, the molecular mechanisms of hypoxia-driven transcriptional activation mediated by Sp1 are diverse and complex, as summarized in this review. To date, the details of these mechanisms are not fully understood. Moreover, the relative biological importance of each Sp1-dependent transcriptional regulation mechanism compared to that of HRE-dependent mechanisms is largely obscure. For instance, hypoxic induction of the *VEGF* gene is a typical example of HRE-driven expression. However, this gene can also be activated in response to hypoxia via activation of Sp1 [28,32]. Future investigations will uncover the answers to these intriguing issues.

To date, a number of small-molecule inhibitors that target and inhibit HIF activity have been reported and exploited [57]. Multiple therapeutic trials targeting HIFs are currently ongoing and await evaluation [74,75]. It was revealed that targeting both HIFs and other molecules, such as metabolic enzymes that are specifically activated in cancer cells, improves therapeutic efficacy compared to cancer treatment targeting only HIFs [74]. A more detailed understanding of the molecular mechanisms underlying the adaptive response of cancer cells to hypoxia is expected to result in more efficient strategies to target tumor hypoxia. Moreover, it is known that oxygenation levels of normal mammalian tissues range from 2% to 9% [1], which can be close to hypoxia levels within tumor tissues. Thus, it is possible that the HIF-targeting strategy, which is expected to inhibit both HRE-dependent and some Sp1-dependent transcriptional activation mechanisms, will result in harmful side effects to cancer patients. One approach to overcome this problem may be to target the more severe hypoxic conditions specific to cancer tissues. Sp1 may predominantly mediate transcriptional activation under very severe hypoxia conditions, as described in Section 3.3. Indeed, it was recently revealed that suppression of the highly synergistic activation of *ICAM1* in CCC cells exposed to severe hypoxia with a limited supply of LCFA leads to the inhibition of tumor growth [50]. Thus, targeting transcriptional regulation that is specifically activated under severe hypoxia conditions may be of clinical benefit. However, as in the case of HIF-targeting strategy, Sp1 inhibition is expected to lead to severe side effects, as this transcription factor mediates many physiologically-important transcriptions. Furthermore, concerns exist regarding the limited tissue diffusibility of drug compounds, because a severely hypoxic tumor area will be poorly vascularized. Future investigations into Sp1-driven transcriptional regulation in response to hypoxia will lead to a greater understanding of the molecular mechanisms underlying the adaptive responses of cancer cells to a harsh tumor environment. These advances will be followed by the identification of novel and promising strategies to treat aggressive cancers.
